# Beyond the Grind: The Intercultural Challenges and Cohesion Efforts in MiLB

**DOI:** 10.3389/fpsyg.2025.1511986

**Published:** 2025-04-11

**Authors:** Claire Sandman Malcomb, Emily M. Zitek, Samuel Grossman, Benjamin Parris

**Affiliations:** School of Industrial and Labor Relations, Cornell University, Ithaca, NY, United States

**Keywords:** baseball, multicultural, intercultural, diversity, inclusion, language, sport psychology

## Abstract

Minor League Baseball (MiLB) comprises players with a wide variety of backgrounds, including many born in the United States and Latin America, and these players spend years trying to work their way up to the Major League level. This paper describes a qualitative study that explores people’s experiences in MiLB, focusing on the challenges that players face and how these challenges might differ for players of different backgrounds. We interviewed 18 MiLB players, nine coaches, and two team education coordinators. Using a thematic analysis technique, we learned that the language barrier seemed to produce problems for the young players, especially those who spoke only Spanish and could not fully benefit from the coaching. Also, some interviewees talked to us about the challenges of intergroup relations, where cliques can form and some players may face biases from coaches. These results suggest that MiLB is challenging for everyone, but there are unique challenges for international Latino players and other minorities. However, we also learned that many teams are trying to solve these issues by offering language classes and other programs. Notably, many interviewees reported positive attitudes toward diversity. Ultimately, we found that while all players experience the same grind trying to make it to the Big Leagues, some players also experience unique and additional barriers to succeeding. We contribute to the broader conversation on the role of cultural diversity in sports.

## Introduction

Baseball has long been considered America’s pastime, but the reality of that may be shifting as roughly 29% of Major League Baseball players are international players (Lapchick, 2023). As the baseball field seems to become more diverse, the experiences of minority players remain underexamined. In the current work, we explore (1) the experiences of Minor League players with a particular interest in how players from diverse backgrounds may experience unique barriers, and (2) potential solutions to advancing equity and inclusion in baseball. We aim to contribute to a broader conversation on the role of cultural diversity in sports.

### The MiLB context and predictors of success

Most baseball players dream of playing Major League Baseball (MLB), but getting there is a long, difficult, and constant grind. After signing with an MLB organization, players usually start in Minor League Baseball (MiLB), aiming to make it to the Majors. Many players remain in the Minors for 4–6 years (Gaines, 2013), earning meager salaries while playing in these lower levels. When players make it to the Majors, their pay improves dramatically, but around just 10% of MiLB players get there (More Than Baseball, 2022).

To get to the Majors, an MiLB player must develop into an excellent pitcher or hitter who can compete against the best. The athlete has to stay motivated throughout the long and arduous process of advancing through the Minors. For many players, this involves years of low pay, unappealing housing and food options, frequent travel, needing to move and adjust to new teams, and searching for a job in the offseason while also having to stay in shape (see More Than Baseball, 2022).

Both physical and psychological factors are significant predictors of success in professional baseball (Smith and Christensen, 1995). What might predict success in MiLB? Sport research has shown that more (v. less) successful athletes are more likely to have physical advantages like height and excellent vision (Epstein, 2013), as well as psychological advantages like greater self-confidence (Jekauc et al., 2023; Lochbaum et al., 2022), better emotion regulation (Tamminen and Kim, 2024; Wagstaff, 2014), and more grit (Apró et al., 2024). Moreover, athletes are more likely to perform better if they feel like their team is cohesive and inclusive (Carron et al., 2002; Malcomb and Zitek, 2025). Athletes are also more likely to succeed with more resources and better coaching (Berry and Fowler, 2021; Tompsett and Knoester, 2022).

The above factors and many others should predict good performance in MiLB, but the likelihood of having high confidence, the ideal emotional states, feelings of inclusion, good coaching, plentiful resources, and more differs for different athletes. There is a lot of diversity among players in MLB and its pipeline (Lapchick, 2023; Malcomb and Zitek, 2025), and their social identities are salient to them (Zitek et al., 2024). There are US-born players of different ethnicities, some of whom played college baseball first and some of whom were drafted straight out of high school (McCue et al., 2019). There are also many international players from various countries who can sign at just 16 years old. About 35% of MiLB players come from Latin American countries/territories such as the Dominican Republic, Venezuela, and Puerto Rico (Malcomb and Zitek, 2025). The international Latino players might first play in the Dominican Summer League, but they soon move to the United States to join their next MiLB team (Ruiz et al., 2020). Players with different backgrounds could have vastly different experiences in the Minors.

While all MiLB players likely face some challenges, international Latino players, Black players, and others who are not White Americans might face additional challenges. Past research from a variety of sports suggests that athletes from minority groups have additional barriers to success. First, athletes from minority groups tend to report a lower sense of belonging within their team or organization (Malcomb and Zitek, 2025). Second, athletes who move to a new country often face acculturation stress, as they aim to learn the language and adjust to their new environment’s norms, customs, and values (Ryba et al., 2016; Schinke et al., 2016). Third, athletes from minority or subordinate groups often experience bias in many forms (Gurgis et al., 2022). Athletes are judged based on stereotypes related to their race, ethnicity, appearance, gender, and nationality (Eagleman, 2011; Foy and Ray, 2019; Peña, 2025; Sartore and Cunningham, 2006; Stone et al., 2012; Stone et al., 1997). Moreover, evaluators such as coaches and referees sometimes treat athletes who share their same identity more favorably (Cunningham et al., 2013; Parsons et al., 2011; Thrane, 2025), such as when a White coach gives more playing time to a White player (Zhang, 2017). This can be especially problematic for minority athletes if they are dissimilar from the decision makers in their sport. And when athletes experience lower belonging, acculturation stress, knowledge of stereotypes, and/or biased treatment from coaches and others, this can harm their mental health (Choy et al., 2021), physical health (Gonzalez-Guarda et al., 2021), performance (Gentile et al., 2018; Krendl et al., 2012; Stone et al., 1999), and desire to participate (Bevan et al., 2021).

## Current research

In the current work, we conducted a series of interviews with MiLB players and coaches to understand their experiences and learn more about potential barriers to success for the players and the practical ways MLB could mitigate these barriers. Following our primary data collection, we also interviewed two MLB education coordinators to learn more about the programs being implemented by MLB teams to help alleviate key barriers. Coding and analysis were conducted following guidelines for thematic analysis (Braun and Clarke, 2006; Braun and Clarke, 2021; Braun et al., 2016). Initially emphasizing an experiential approach, we utilized coding as an analytic tool to create a hierarchical structure of codes and initial themes. We then shifted to a more critical approach and further refined our themes through iterations between the data, literature, team discussions, and our goals in the research project.

## Interviews with MiLB players and coaches

### Participants and procedure

During the baseball season in the summer of 2019, we conducted 27 semi-structured interviews with players (*n* = 18) and coaches (*n* = 9) from six different MiLB teams (see Table 1 for detailed demographics). Our contact at MLB headquarters connected our research team to individual MiLB clubhouses. We then coordinated with each team to schedule interviews with players and coaches who agreed to participate. Most interviews were in-person at the clubhouse, but a few were via phone. Interviews were audio recorded with consent; otherwise, detailed notes were taken. No one on the research team was fluent in Spanish, so if an interviewee spoke only Spanish, the clubhouse provided a translator.

**Table 1:** Demographic information for interview participants.

		Players	Coaches	Total sample
Age	Average	22.89	48.0	31.26
	Range	20–28	29–62	20–62
Nationality	# of US born	14	6	20
	# of Non-US born	4	3	7
	Countries represented	Canada, Dominican Republic, Puerto Rico, United States, Venezuela	Dominican Republic, Jamaica, United States, Venezuela	Canada, Dominican Republic, Jamaica, Puerto Rico, United States, Venezuela
Teams	# of teams represented	5	6	6
	Levels of Minors represented	4 (A-, A, AA, AAA)	4 (A-, A, AA, AAA)	4 (A-, A, AA, AAA)
Interviews	Sample size	18	9	27
	# recorded	18	8	26

In collaboration with our MLB contact and his staff, we developed guiding interview questions to help us understand the experiences of MiLB players (for the complete list of questions, see Table 2). In these interviews, we asked players to tell us about their backgrounds, their main challenges in MiLB, the interpersonal dynamics on their teams, and how players’ experiences might differ depending on their backgrounds or clubhouses. We asked coaches similar questions, plus questions about onboarding players and their coaching methods. After conducting all 27 interviews, we transcribed the recorded interviews using Otter.ai software and manually reviewed the transcripts for accuracy.

**Table 2:** Guiding interview questions.

**Interview questions for players**
What was your experience in baseball before signing your first contract? *(ex. Travel ball, house league, high school ball, college ball, national team, etc.)*
What are some of your main challenges as a Minor League player? *(ex. Financial, being away from home, nutrition, etc.)*
Do you feel a sense of cohesiveness on your team? If there are cliques on the team, how are they formed?
What have been the successes and challenges that you have faced moving from one level to the next in Minor League Baseball?
If you have played in different organizations or different levels of the Minors, please discuss the factors that vary from team to team. *(Coaches, facilities, towns, etc.)*
How do you feel the Minor League experience differs for players from diverse backgrounds?

**Interview questions for coaches**
*If the coach has played* Please discuss your transition from one level to the next when you were a player. What were the challenges that you faced?
What are some of the struggles that you see the players on your team facing? (*On-field or off-field*)
Are there cliques on the team? How are the groups formed?
Please discuss the process of onboarding new players onto the team, whether at the beginning of the season or as players are acquired, sent up, or sent down.
How are you prepared to manage and work with a team of players from diverse backgrounds and with unique personalities? Does your organization provide you with training and support to face this challenge?
How do you feel the Minor League experience differs for players from diverse backgrounds?

### Data familiarization and initial coding

Members of the research team independently familiarized themselves with the data by reading the transcripts, summarizing the data, and noting potential codes. The research team then met to develop an initial codebook, iterating between the data, our individual notes, and group discussion. We next systematically coded the interviews and began organizing codes based on commonalities among the experiences of the players and coaches, generating initial themes. Our coding and initial theme generation at this stage were primarily descriptive, summarizing the experiences and opinions of our participants (see Table 3 for data extracts illustrating prominent codes and initial themes).

**Table 3:** Data extracts illustrating prominent codes and initial summarizing themes.

Summarizing themes *(Details on included codes)*	Data extracts representative of codes
**Resource constraints** *Challenges related to finances, nutrition, travel, family, and staying healthy*	“I’d say that the biggest thing for me this year was the travel…we’ll have trips that takes 7 h, 5 h, and you have to drive back, work the night after game, and then have to play the next day. So, just, it messes you up to switch gears a little bit...on the road it’s hard to find the right food because …you are on the bus. So you have to Uber places, it’s definitely a lot harder to get the food that you want, the food that your body might need. There’s been a lot of gas station trips just trying to find any little thing, kinda trying to find nuts or seeds or little fruit cups from there. So yeah, I think the travel and nutrition is just hard to get consistently, consistently good out of those two things.” (player 11)
	“Being able to support and live a comfortable life while playing I think is tough when you get paid what you get paid… it’s hard being away from family and friends. Cuz we are gone all year. Then the time we are home, we have to designate a lot of it to get ready for the next season. Some other challenges, staying healthy is a big challenge, just physically the workload of the schedule. And then being able to perform under the time frame that we have to recover is pretty tough, but I think it’s a part of it. It just comes with the job.” (player 18)
	“I can deal with the shitty fields, hotels, travel schedules, and food. But when I have to get a job just so I can make it through the offseason, it does not sit right with me. Serving steaks should not be my primary focus in the offseason.” (player 12)
	“You’re not making a lot of money. You’re kind of living paycheck to paycheck, kind of even going minus most of the time.” (player 14)
**Mental toughness** *Psychological challenges in professional baseball*	“It’s tough because…baseball is a failure sport. You know, you get like, 3 hits out of 10 times and you are the best of the world. But you are a failure seven more times. So you are dealing with that. And those players, you know, it’s tough. I’ve been through that too as a player. And it’s challenging every day to keep your mind tough and just keep moving forward. Even though you went 0 for 4 for the night or 0 for 5 with three strikeouts. Tomorrow’s another day, you know, so you have to be prepared for that day, day by day. So mental part is tough. And I think that’s why baseball is more mental sport than anything else. Because they all got talent, you know, they all that’s the reason why they’re here. …it’s it’s more mental or whoever’s been tougher mentally is going to forward quicker. And that’s the way I see it” (coach 22)
	“Some of the struggles that I see like some of those guys, they on a process to learn how to how... they need to handle themselves when they come to be a professional baseball player, because baseball, not every time things gonna go your way. So you had to keep your mind strong. When those times come when you fail, how to stay strong. And think about, okay, I gotta have a one more day to perform and be a better player...that’s one of the struggles that I see that those guys that... they need to be mental tough.” (coach 20)
	“Some of the setbacks are when you are not doing so well. And you have to keep digging and keep trucking along when, you know, you are not making a lot of money. There’s other things to be doing. So just really sticking through it. The grind, as we all call it, is definitely, it’s rewarding in the end.” (player 16)
**Language and cultural barriers** *Additional challenges unique to non-English speakers and international players*	“…coaches pretty much were going to be American, and for them to get the fullness or the full benefit of the coaches, they had to understand English.” (coach 21)
	“…since I work as the pitching coach and work with the pitchers, being able to hit a group message and get that translated, which that does take some extra time, I had to have somebody who’s going to translate, because I can only speak enough to be able to help guys, mainly just pitching types of delivery and stuff. But as far as having a conversation, that’s what makes it a little bit difficult for me. And then having to have somebody translate and go through the filter of that, without my message directly impacting them and getting across the way I want it to get across, having to rely on somebody else that, that makes it a little bit different.” (coach 19)
	“They do they make sure that the guys from other countries are like going to English class and kind of learning our culture, so that they can adapt because obviously, like, we, I feel like we have an easier time adapting to them then they have or they are having to adapt to us, and our culture of how we do things in like America. But I think for the most part, like they do a pretty good job” (player 6)
	“I think going over in to Korea, and being the foreigner really opened up my eyes to some of the challenges that, that, you know, other people might have, I think, you know, like I said, the Latin players coming over, especially at such a young age…I went over to Korea when I was 26 years old, and I, I struggled with the language barrier, and just trying to figure things out and, and I had a translator, and these guys, you know, they do not have a translator, and they are kind of, you know, fending for themselves and trying to figure out how to live in America. And I think that can be extremely difficult for sure. And I think I gained a lot of respect for those guys. And the challenges that they have.” (player 17)
**Ingroup and outgroup effects** *Cliques formed by social identity groups; coaches’ preference for certain players; discrimination experienced*	“I’ve seen sometimes they prefer the white guys over the Latin guys… Sometimes, sometimes when, when a Latin guy does something bad… like they’ll yell at them. And then when American guy does the same thing they, like, they’ll do not do anything about it.” (player 8)
	“It’s definitely a double-edged sword, certain things that you can get away with as a White player that you are not allowed to get away with as a Black player.” (coach 24)
	“Um, I would say there are some cliques sometimes between language barriers and things like that, because we have some guys maybe know like 20 words in English. So it’s kind of like they got to stick with the guys that know both. And some, some of us do not know any words like in Spanish. So just kind of stick with the guys that do not know any in Spanish.…” (player 1)
	“...as we get into pro ball, you know, those were the kinda cliques that started….you know it might be more of the Spanish speaking players… they would hang more together, obviously, you know the language, it’s just easier to communicate within their own language….” (coach 25)
	“So, if there’s a coach, who’s Dominican and can relate to the Dominicans on the team, or like the people with different backgrounds... I think they are more inclined to really like, take... to help them out in a way in a sense, like, like give them extra work and really try to teach them rather than um like people like of different backgrounds.” (player 9).
**Team cohesion and shared goals** *Efforts to learn from one another, grow together, and build team chemistry*	“For instance, a lot of the college guys, I mean, we all get along together, I think the one thing that like separates a little bit is, you know, if there’s like Dominicans on the team, to Americans, I think that’s, there’s like a little bit of a like a, like a separation between the two. But everyone kind of gets along. And I.. it kind of feels like a team, rather than you are playing for individual self, which I definitely like, love, playing it on a team rather than a bunch of individuals who are just trying to outperform each other... does not really feel like that, which is good.” (player 9)
	“Just because everybody knows that we are all trying to make it to the Big Leagues. And there’s not, there’s not too many spots up there. So we know that we are competing for the spot, but at the same time it helps you, I’ve noticed just with myself, whenever I try to help somebody else, it helps me get a better understanding of what I’m trying to do. If I’m trying to, teaching, if I’m showing somebody maybe that they need to catch the ball more out of front, I’m helping them out. But at the same time, I’m reminding myself that I need to do the same thing.” (player 11)
	“We tell them a lot of times, ... you win the game in the clubhouse before you before you even go out and feel the team chemistry and stuff like that… ... we try to get our Latin American players as well as our English players to interact with each other, and, you know, learn about each other’s countries and upbringings and stuff like that. So and I’m sure in the clubhouse in there where they develop team chemistry they do that.” (coach 27)
	“I would say it’s easier for guys who speak the same language to, you know, gravitate towards each other just because it’s easier communication. I mean, the [team name] are doing a good job though, they have like English class Spanish class pretty much every week. So they are doing a good job to try and mix those groups up. But I think language is the main one… I mean, guys show interest in like, trying to learn Spanish like here, I would say it’s probably like half ‘n’ half. So you got like guys, that speak only English trying to learn Spanish, Spanish guys trying to learn English. So that kind of builds that team chemistry as I was talking about.” (player 7)
	“It’s kind of cool knowing people who come from other countries, other cities, and getting to know each other and share being teammates here…they are looking at the same goals, just to make it to the Big Leagues” (player 13)
**Positive attitudes toward diversity** *Coaches adapting to the needs of players; acknowledgement of the importance of support for players from diverse backgrounds*	“I was actually talking to a guy about this yesterday, if I worked with the Latin pitcher different than, say, a college pitcher. And I said yeah, I mean sure you do. Because the level that they are, they are on it, we are pitching at the same level, but as far as... typically, they are a little bit younger. So working with them, and giving them a more simpler task, because most of them did not go to college. So, they did not have 4 years to be able to pitch and learn what they needed to do. So, keep it simpler.” (coach 19)
	“And then we try and teach English to the guys like during the game like… we try to like give them a phrase every day to try and learn. So it’s just been fun. I’ve had fun with just being able to learn their culture, how they work, because it’s a totally different world, where they are from.” (player 6)
	“There’s a lot of players from Latin America. And for me, it’s been really cool to get to know them and understand their lives away from baseball, as well as in baseball. It’s something I would never have experienced before. And I’ve gotten to know them personally and really enjoyed their friendship…camaraderie, throughout baseball, it’s cool, it’s opened my eyes and kind of opened my world a little bit more than it would have been before.” (player 16)
	“Our coach spoke both languages. So he’d say everything in English, then he would say everything in Spanish right after. So both groups can understand it. And some guys I’d say do not speak English, or do not speak Spanish. And that was really helpful to have everybody on the same page. Again, it was annoying if you had to repeat yourself, but that’s something that they do. Meals will be like... try to cook what people prefer, you know, there will be like a large percentage of guys from the Dominican Republic…they made meals that were like Dominican meals. That was cool. Just inclusion, inclusive stuff like that.” (player 4)
	“Having like a language barrier between some people can kind of can make it like people will group together but I think like you try and learn the other languages and I think that that helps. And they have like in Florida, they’ll have us take, like, Spanish classes, where you can try and like kind of break that barrier….Well, here… the Spanish guys have to still take English class and we do not have to. But I think I think obviously a lot of them want to learn more because it helps to stay in America and be able to, like, be on their own. So I think that’s it’s definitely like a motivating factor for them. Then for us, it’s motivating so you can [hangout with] your friends and be able to talk to them more.” (player 2)
	“I feel like if you come in here with a mindset that like it’s different, and like you do not understand anything, like you are gonna be in trouble. But if you have an open mind, and you just you work to understand them and understand where they are coming from, that it just makes you adapt and get used to it.” (player 3)
	“I think it only hurts people if they are not open to learn or trying to get on the same page. That would be it... but I took Spanish in high school, and I’m not fluent, but I know enough to communicate on the field, that it’s kind of second nature for them... But I know enough Spanish where we can communicate on the field… You can just learn enough either way, which is not asking much, then it’s really not a hindrance. (player 5)

#### Summary of data

Like in the report by More Than Baseball (2022), many interviewees spoke about the low pay, the difficulty obtaining additional employment in the offseason, nutrition and travel hardships, and the mental challenges associated with playing a highly competitive sport. Another common challenge brought up by players was the language barrier between the Americans and international Latinos. Our interviewees described how the language barrier led cliques to form based on language and that some players had trouble communicating with their coaches. Although the language barrier seems challenging for everyone, it might produce additional hardships for international Latino players, who, for example, may not be able to understand coaching instructions given in English by their mostly American coaches.

Some players and coaches shared that players were sometimes treated differently based on their identities. Sometimes the interviewees considered this differential treatment to indicate unfair bias against minority groups. For example, one player said, “Sometimes when a Latin [American] guy does something bad, like, they’ll yell at them. And then when an American guy does the same thing they, like, they do not do anything about it” (player 8). In addition to their nationality, a player might experience bias due to race. For example, one coach said, “[There are] certain things that you can get away with as a White player that you are not allowed to get away with as a Black player” (coach 24). Moreover, consistent with other research on same-identity favoritism (Zhang, 2017), other interviewees hinted that coaches might be biased toward players who are similar to them. Some coaches “give them extra work and really try to teach them rather than people of different backgrounds” (player 9). If coaches favor people of the same identity, then this will disproportionately affect minority players since most coaches are White Americans. In sum, bias toward minority players is a problematic barrier that seems to work in conjunction with systemic barriers.

The differential treatment was sometimes considered necessary due to the players’ different backgrounds. For example, one coach mentioned that he keeps it simpler for Latin American players “because most of them did not go to college. So, they did not have four years to be able to pitch and learn what they needed to do” (coach 19). Other interviewees mentioned that it is helpful for players to have a coach who shares an identity with them and therefore understands their perspective. For example, when a Latin American player has a Latin American coach, that can be highly beneficial, because, as one player said, “a coach who’s Dominican…can relate to the Dominicans on the team” (player 9). Unfortunately, one interviewee pointed out that Black coaches are rare and therefore Black players might not have anyone who can relate to them in that way.

Though the interviewees acknowledged that cliques seemed to form naturally based on language and cultural differences, along with other differences in backgrounds, many interviewees held an overall positive attitude toward the team’s diversity. For example, one player said that he enjoyed the formal language classes offered by the organization, and another player talked about how the players informally helped each other learn Spanish and English to enhance their ability to communicate. Players also spoke about enjoying learning about different cultures, with one saying that it “kind of opened my world a little bit more than it would have been before” and “it’s just been fun.” Consistent with this, other research has revealed that Latin American baseball players view the cultural transition as a positive experience (Gentile and Arth, 2022). In short, despite the challenges, many players really liked the multicultural environment in MiLB.

## Interviews with Education coordinators

To follow up on our initial insights, we conducted unstructured interviews with two MLB education coordinators between 2019 and 2021. Many MLB organizations recognize that international players face extra barriers, and they have therefore hired education coordinators to facilitate various aspects of international players’ transition to the United States. The education coordinators work with players at all levels in baseball, often spending a lot of time at the Dominican Summer League. The education coordinators also oversee the language classes. Thus, a goal of these interviews was to learn more about the challenges faced by players of different backgrounds, but we centered much of our conversation on the initiatives teams were putting into place to help address these issues.

One education coordinator commented that teams had realized in the past 10 years that there was a competitive disadvantage in underfunded or non-existent education programs and had therefore started increasing their resources. At the time of our interviews, language programs were enacted in a variety of different ways across the various organizations. For example, while almost all organizations offered some sort of English courses for Spanish-speaking players, some organizations also offered Spanish courses for English-speaking players. Moreover, these courses might cover more than just language. One education coordinator said, “When I first started, there was more of a focus on practical English, but more recently we have transitioned to incorporate learning personal and professional skills.” Beyond the language barrier and challenges with acculturation, the education coordinators pointed out that a lack of formal education can also be an added challenge for some Latin American players. The international Latino players can sign with a team at a younger age than the Americans, which can lead them to get pulled out of school earlier. Also, some Latin American countries do not have as strong of education systems as others, and therefore there are differences within the international Latino players. The education coordinators keep this in mind as they work on their programming. One education coordinator told us that, at the time of these interviews, most MLB organizations were instituting programs to help the young international players earn their GEDs, as they recognized the importance of education.

### Theme development and refinement

Following these interviews, the research team reviewed the coding and initial theme generation from the player and coach interviews, integrating findings from the interviews with education coordinators. Insight from the coordinators mirrored many of the key issues brought up by players but introduced a solution-oriented lens through which we thought about the barriers to success. Following recommended thematic analysis practices (Braun and Clarke, 2006 Braun and Clarke, 2021), our analysis approach was both descriptive and interpretive from this point on, allowing us to understand more latent patterns in the data. After collecting, coding, and summarizing the data, the first two authors worked to further develop themes, iterating between the data, coding and initial themes, writing, discussions with co-authors, and previous literature.

#### Theme 1: The same grind, but different struggles

As noted previously, there are many structural barriers faced by players in MiLB: the low pay, difficulty getting a job in the offseason, intense travel schedules, difficulty getting the proper nutrition, and stress of competing at a high level. Players and coaches alike conceptualized these hardships as just part of “the grind,” commonly used in sports to describe the long, difficult, and demanding season. Specifically in the context of the Minors, these hardships are both expected and accepted as “part of it” while they are all trying to make it to the Majors.

More broadly, the impact of some of these structural barriers on players differed for players from different backgrounds. For example, several interviewees mentioned that younger players may struggle through learning how to live on their own for the first time, while the players who came to the Minors after playing in college may have an easier time with the adjustment. Moreover, some MiLB players have large signing bonuses and therefore may not have as many problems with the low salaries.

The barrier most noted by our participants was the language barrier. Many players can only speak either English or Spanish when they start in MiLB, and suddenly they need to interact with people who speak another language in this competitive, high-stakes environment. As one coach said, though players are “kind of all in the same boat, [MiLB could] definitely be more challenging for a person that does not speak English, or like the native language” (coach 23).

The language barrier was discussed in terms of creating social and task-related challenges. For social challenges, players described how language differences created cliques on the teams, such that Spanish speakers would hang out with each other and English speakers would hang out with each other. The tendency to interact more with similar others (homophily) is consistent with other research on intergroup relations (Carey et al., 2022).

While these cliques and the language differences between players did not seem to bother any of the interviewees, the task-related challenges brought about by language differences seemed to have a larger impact. More specifically, “…coaches pretty much [are] going to be American, and for [players] to get the fullness or the full benefit of the coaches, they [have] to understand English” (coach 21), putting Spanish-speaking players at a disadvantage for developing their skills and learning from their coaches. Differential access to quality coaching could partially explain why American players progress through the Minors more easily than international Latino players do (Malcomb and Zitek, 2025).

Some interviewees also brought up biases that could hinder the advancement of international Latino and other minority players. Some players mentioned that some coaches might get more frustrated with certain individuals than others and might “yell at” the international Latino players or not let a Black player “get away with” something. One education coordinator also mentioned that there are sometimes issues with stereotypes and biases on the part of decision makers, which can affect which players get promoted, retained, or cut. Moreover, the coaches seemed to have different expectations for different athletes, which was understandable since the athletes had very different starting points. However, if coaches have lower expectations for some athletes based on their backgrounds, they might give them worse feedback and fewer opportunities, potentially hindering their development (Solomon et al., 1996; Weaver et al., 2016).

#### Theme 2: Breaking barriers in the dugout, building chemistry on the field

Though several players and coaches mentioned experiences of discrimination, many of our participants also spoke of the dissimilar treatment across players as not inherently negative; coaches recognized that different players had varying needs, and they tried to coach them differently, such as, for example, giving different instructions to an American player who had attended college vs. a young international Latino player who had not. Giving individual consideration is an important element of good leadership and can bring about good performance in sports (Mach et al., 2022). That said, coaches certainly need the ability to communicate with players to be able to give them the individual attention they deserve.

Given the challenges due to the different player backgrounds, the language barrier, and the intergroup dynamics, it might be important for coaches to get additional training. They can learn how to reduce cliques to help team cohesion and performance (Eys et al., 2009), and how to find a coaching style that fits their players. Past research has indicated that autonomy-supportive coaching behavior can help buffer against athletes’ acculturation stress (Morela et al., 2019). One education coordinator agreed with our other interviewees that getting players from different countries to be a cohesive unit can be challenging. However, some players have done a great job promoting inclusive environments on their teams. This education coordinator strongly endorsed the importance of not just hiring *bilingual* coaches but also hiring *bicultural* coaches who can better relate to the Latin American players and can therefore help them progress through the Minors.

These positive aspects of recognizing and adapting to the unique needs of diverse players reflected a broader ideology of multiculturalism within baseball, broadly defined as recognizing and celebrating the meaningful differences between different racial, ethnic, and cultural groups (Rosenthal and Levy, 2010). Importantly, while players and coaches alike acknowledged the additional barriers of learning a new language and culture for many international MiLB players, the underlying sentiment was not one of assimilation to an American culture (i.e., an expectation that international players will simply learn English and “fit in” to American culture). Rather, many seemed to view this context of baseball through an intercultural lens whereby everyone was actively learning each other’s cultures (Yogeeswaran et al., 2020). For example, while players and coaches acknowledged how important it is for teams to provide English classes for the international Latino players, many also pointed out the importance of having English-speaking players learn Spanish.

## Discussion

MiLB is hard, with the low pay, grueling schedule, complicated intergroup dynamics, and long road to the Majors. But as we learned in our interviews, some players have extra challenges. For example, the international Latino players move to a new country, learn a different language, face acculturation stress, and possibly experience bias and frustration from their mostly English-speaking coaches. This might explain why American players progress through the Minors more easily than international Latino players do (Malcomb and Zitek, 2025).

Despite the many challenges that were mentioned about MiLB in general and the diverse environment in particular, some players also noted benefits of the experience. Specifically, it seems that many players have a positive attitude toward diversity. They like meeting people from different cultures and learning new languages. According to theory and research on the contact hypothesis within and outside of sports, interacting with outgroup members and working together to achieve a common goal can help improve intergroup relations and reduce prejudice (Allport, 1954; Ellison et al., 2011; Graber and Zitek, 2022; Lowe, 2021; Mousa, 2020; Pettigrew and Tropp, 2006; Zhou et al., 2019). Positive feelings about the outgroup were already apparent in our interviews, and intergroup relations should improve further as these players continue in baseball.

MLB has been working to address the challenges faced by MiLB players, as evidenced by hiring education coordinators, the development of language classes, the increase of MiLB players’ pay, and more. As new initiatives are implemented that focus on removing barriers for minority players and generally improving intergroup relations, we are optimistic that MiLB players will be enthusiastic participants given their current positive views on diversity. MLB can better leverage its diversity if all players feel like their team is cohesive and inclusive, as these beliefs relate to greater perceived performance (Malcomb and Zitek, 2025).

Finally, although many interviewees focused on interactions between American and international Latino players, some race-related issues were also mentioned. For example, some of our interviewees said that there may be different expectations and standards for White vs. Black players. This is a crucial issue for league leaders to consider as they work to increase the number of Black players in MLB (see Castrovince, 2023).

### Limitations and future directions

Our study produced interesting insights but also had some limitations affecting interpretations and generalizability. First, we interviewed just 18 players, nine coaches, and two education coordinators. We could not interview players from all organizations or levels of the Minors, we did not have any Asian or Asian-American participants in our sample, and we had just six nationalities represented. Players from these other groups may have had different experiences. Second, though players and coaches spoke about their own experiences, we cannot say for sure how the barriers reported and the attempts to correct them (e.g., the language programs) affect player and team performance. Future research could further study this, perhaps by incorporating performance statistics. Finally, future research should explore the intersection of identities such as nationality and race/ethnicity and how the intersection might affect player outcomes.

## Conclusion

In conclusion, through interviews with MiLB players and coaches, we learned about the challenges of the Minors. We found evidence that minority players face additional barriers to success beyond the challenges faced by White and/or American players. While some players talked about individual-level biases, another prevalent contributor to inequality was a set of structural barriers. For example, because most coaches speak English, the Spanish-speaking players seemed disadvantaged in getting instructional training and forming relationships with coaches. Despite the challenges, many interviewees had positive attitudes towards the growing diversity within baseball and the initiatives that aim to overcome some of these structural barriers, such as the language classes. Given this prevalent positive view of diversity, we hope that players and coaches will choose to participate in other initiatives put forward by MLB and its associated teams as they work to ensure that all players have an equal opportunity to succeed.

## Data Availability

The data presented in this article are not readily available to maintain the privacy of identifiable information within the data. Requests to access the datasets should be directed to the corresponding author.
